# CoDP: predicting the impact of unclassified genetic variants in *MSH6* by the combination of different properties of the protein

**DOI:** 10.1186/1423-0127-20-25

**Published:** 2013-04-28

**Authors:** Hiroko Terui, Kiwamu Akagi, Hiroshi Kawame, Kei Yura

**Affiliations:** 1 The Graduate School of Humanities and Sciences, Ochanomizu University, 2-1-1 Otsuka, Bunkyo, Tokyo, 112-8610, Japan; 2 Division of Molecular Diagnosis and Cancer Prevention, Saitama Cancer Center, 818 Komuro, Ina, Kitaadachi, Saitama, 362-0806, Japan; 3 Center for Informational Biology, Ochanomizu University, 2-1-1 Otsuka, Bunkyo, Tokyo, 112-8610, Japan

**Keywords:** HNPCC, In silico, Lynch syndrome, Mismatch repair, MSH6, Unclassified variants

## Abstract

**Background:**

Lynch syndrome is a hereditary cancer predisposition syndrome caused by a mutation in one of the DNA mismatch repair (MMR) genes. About 24% of the mutations identified in Lynch syndrome are missense substitutions and the frequency of missense variants in *MSH6* is the highest amongst these MMR genes. Because of this high frequency, the genetic testing was not effectively used in *MSH6* so far. We, therefore, developed CoDP (Combination of the Different Properties), a bioinformatics tool to predict the impact of missense variants in MSH6.

**Methods:**

We integrated the prediction results of three methods, namely MAPP, PolyPhen-2 and SIFT. Two other structural properties, namely solvent accessibility and the change in the number of heavy atoms of amino acids in the MSH6 protein, were further combined explicitly. MSH6 germline missense variants classified by their associated clinical and molecular data were used to fit the parameters for the logistic regression model and to assess the prediction. The performance of CoDP was compared with those of other conventional tools, namely MAPP, SIFT, PolyPhen-2 and PON-MMR.

**Results:**

A total of 294 germline missense variants were collected from the variant databases and literature. Of them, 34 variants were available for the parameter training and the prediction performance test. We integrated the prediction results of MAPP, PolyPhen-2 and SIFT, and two other structural properties, namely solvent accessibility and the change in the number of heavy atoms of amino acids in the MSH6 protein, were further combined explicitly. Variants data classified by their associated clinical and molecular data were used to fit the parameters for the logistic regression model and to assess the prediction. The values of the positive predictive value (PPV), the negative predictive value (NPV), sensitivity, specificity and accuracy of the tools were compared on the whole data set. PPV of CoDP was 93.3% (14/15), NPV was 94.7% (18/19), specificity was 94.7% (18/19), sensitivity was 93.3% (14/15) and accuracy was 94.1% (32/34). Area under the curve of CoDP was 0.954, that of MAPP for MSH6 was 0.919, of SIFT was 0.864 and of PolyPhen-2 HumVar was 0.819. The power to distinguish between pathogenic and non-pathogenic variants of these methods was tested by Wilcoxon rank sum test (*p* < 8.9 × 10^-6^ for CoDP, *p* < 3.3 × 10^-5^ for MAPP, *p* < 3.1 × 10^-4^ for SIFT and *p* < 1.2 × 10^-3^ for PolyPhen-2 HumVar), and CoDP was shown to outperform other conventional methods.

**Conclusion:**

In this paper, we provide a human curated data set for MSH6 missense variants, and CoDP, the prediction tool, which achieved better accuracy for predicting the impact of missense variants in MSH6 than any other known tools. CoDP is available at http://cib.cf.ocha.ac.jp/CoDP/.

## Background

Lynch syndrome (MIM: #120435, #609310), also known as Hereditary Non-Polyposis Colorectal Cancer (HNPCC), is an autosomal dominant disease and the most common hereditary colorectal cancer syndrome [1]. Lynch syndrome accounts for 1-5% of all colorectal cancer (CRC) patients [2-4] and associates with germline mutations in one of the DNA mismatch repair (MMR) genes including *MLH1, MSH2, MSH6* and *PMS2* (MIM: #120436, #609309, #600678, #600259, respectively). MMR gene mutation carriers are at high risks of developing Lynch syndrome associated cancer at colorectal, endometrial, small bowel, stomach, ovary, ureter and hepatobiliary tract. Individuals at high risks can be identified by the use of genetic testing, and appropriate surveillance programs can be provided to prevent cancer development.

Previous studies reported that more than 90% of the detectable mutations in Lynch syndrome were found in *MLH1* and *MSH2*[5]. Recent data, however, showed that *MSH6* contributed to about 20% of the mutations [6,7]. In addition, MSH6 shows the greatest frequency (~37 - 49%) of missense variants in the MMR genes, and most of them are currently “unclassified variants” (UVs) [6,8].

*MSH6* mutation carriers tend to develop CRC at the age elder than *MLH1* and *MSH2* mutation carriers and tend to show reduced penetrance [9-12]. These tendencies suggest that family cancer history with an *MSH6* mutation should not be necessarily dense enough to meet the Amsterdam criteria. Furthermore, colorectal tumor from *MSH6* mutation carriers sometimes demonstrates microsatellite instability low (MSI-L) or microsatellite stable (MSS) [13], or normal staining pattern of immunohistochemistry (IHC) for MMR proteins [11]. It is, therefore, important to analyze and integrate all the available data, and the data derived from the use of *in silico* tools for the classification of UVs is one of them.

A number of methods to predict the biological effects of missense variants as pathogenic or genetic have been reported. For Lynch syndrome, SIFT [14], PolyPhen [15,16] and multivariate analysis of protein polymorphisms (MAPP) [17] have been used in general. Predictions using SIFT is based on sequence conservation, while that of PolyPhen is based on sequence conservation plus protein structural features [14-16]. These methods aim to predict the pathogenicity of variants for general proteins and hence they were not tuned to the interpretation of the prediction for a specific protein. MAPP uses the evolutionary variations and scales of six physicochemical properties to evaluate the structural and functional impact of all possible variants [17]. MAPP can be customized for a specific protein. It has been optimized to MLH1 and MSH2 and outperformed SIFT and PolyPhen (MAPP-MMR [18]). This result indicates that the algorithm customized for a specific protein is superior to those applicable to proteins in general. However, the accuracy of prediction by MAPP-MMR is not satisfactory enough for the genetic testing. Hence, improvement in the prediction method is required.

In the field of bioinformatics, especially the field for developing a prediction method out of amino acid sequences, it has been pointed out that the prediction accuracy can be improved by integrating many different prediction methods (*e.g*. [19]). Following this idea, the accuracy of the pathogenicity prediction could be improved by integrating a number of existing methods to predict the biological effects of missense variants. In addition, none of the existing methods directly incorporate the information obtained from the MSH6 protein structure. The three-dimensional structure of MSH6-MSH2 complex with ADP and DNA was already solved [20]. The structural data should contain varieties of information, some of which would be useful for the prediction. The easily obtained information related to the mutation effect to the structure includes the solvent accessibility of amino acid residue and the residue volume change. The mutation of amino acid residue at the surface of the protein are tolerant compared with that in the interior of the proteins, and a small volume change in amino acid residues in mutation inside the protein is tolerant compared with a mutation with a big volume change [21].

We, therefore, optimized MAPP [17] for MSH6 and then integrated SIFT [14], PolyPhen-2 [15] and two properties from protein structure, namely solvent accessibility and the volume change in amino acid residues. We joined these properties on the logistic regression model and compared the prediction performance with MAPP, SIFT, PolyPhen-2 and PON-MMR [22]. The parameter adjustment was done on the data that we gathered from different databases and literature and associated them with one another for this study. The newly developed method achieved the best prediction accuracy, sensitivity and specificity, and can distinguish pathogenic variants from non-pathogenic variants clearly. We named the method CoDP, Combination of Different Properties on MSH6, and made it available at http://cib.cf.ocha.ac.jp/CoDP/.

## Methods

### The dataset of MSH6 missense variants

MSH6 missense variants and their associated clinical and molecular data were collected from the following databases:  InSiGHT  (http://www.insight-group.org/), MMRUV (http://www.mmrmissense.net/), UniProt (http://www.uniprot.org/), dbSNP (http://www.ncbi.nlm.nih.gov/projects/SNP/), NHLBI Exome Sequencing Project (ESP) (http://evs.gs.washington.edu/EVS/), HapMap Project (http://hapmap.ncbi.nlm.nih.gov/) and 1000 Genomes (http://www.1000genomes.org/). A systematic literature search was conducted on PubMed (http://www.ncbi.nlm.nih.gov/pubmed/) to compile unregistered MSH6 missense variants in the databases above. These data were used to assess the *in silico* pathogenicity prediction.

Clinical and molecular data on carriers with missense variants were also collected. The data included the age at the first diagnosis of CRC or endometrial cancer, any affected relatives with Lynch syndrome associated cancer, microsatellite instability (MSI), IHC, segregation study, allele frequency and biochemical functional assay. The biochemical functional assay included the investigations of the following; MMR activity, MSH2 protein interaction, localization, ATP hydrolysis and mismatch recognition. We employed the results of the assay from the literature as is. These clinical and molecular data were used to divide the carriers into one of the following three categories; “likely to be Lynch syndrome (LLS)”, “unlikely to be Lynch syndrome (ULS)” and “unclassified.” LLS is a carrier with pathogenic variant, and ULS is a carrier with non-pathogenic variant. An “Unclassified” carrier has a variant with unknown clinical significance, which is usually called unclassified variant (UV). The division was carried out based on the criteria shown in Table 1. When a carrier fulfilled one or more of the criteria for LLS in Table 1, the carrier was classified as LLS, and when a carrier fulfilled one or more of the criteria for ULS, the carrier was classified as ULS. When the criterion that the carrier fulfilled became important, a sub-numbering system was used, such as LLS-1 for a carrier fulfilling the first criterion of LLS.

**Table 1:** Definition for classification of missense variants in MSH6

**LLS (Likely to be Lynch Syndrome):**	**ULS (Unlikely to be Lynch Syndrome):**
**Fulfill one or more of the following criteria;**	**Fulfill one or more of the following criteria;**
1. Abnormal result of functional assay **AND** [abnormal IHC of only MSH6 **OR** MSI-H]	1. Polymorphism (minor allele frequency ≥.01)
2. Abnormal IHC of only MSH6 **AND** MSI-H	2. Normal result of functional assay **AND** [MSS **OR** normal IHC of MSH6]
3. [Abnormal IHC of only MSH6 **OR** segregation analysis] **AND** fulfill at least two of the following three criteria.	3. MSS **AND** normal IHC of MSH6
a) Family history: More than one affected relatives who were diagnosed as CRC or endometrial cancer under 60 years old and at least in two successive generations.	
b) Proband‘s tumor feature: diagnosed as CRC or endometrial cancer under 50 years old and/or synchronous or asynchronous multiple cancers.	
c) Control allele frequency = .00 (healthy population ≥ 100)	

### Optimization of MAPP for MSH6

**Figure 1:**
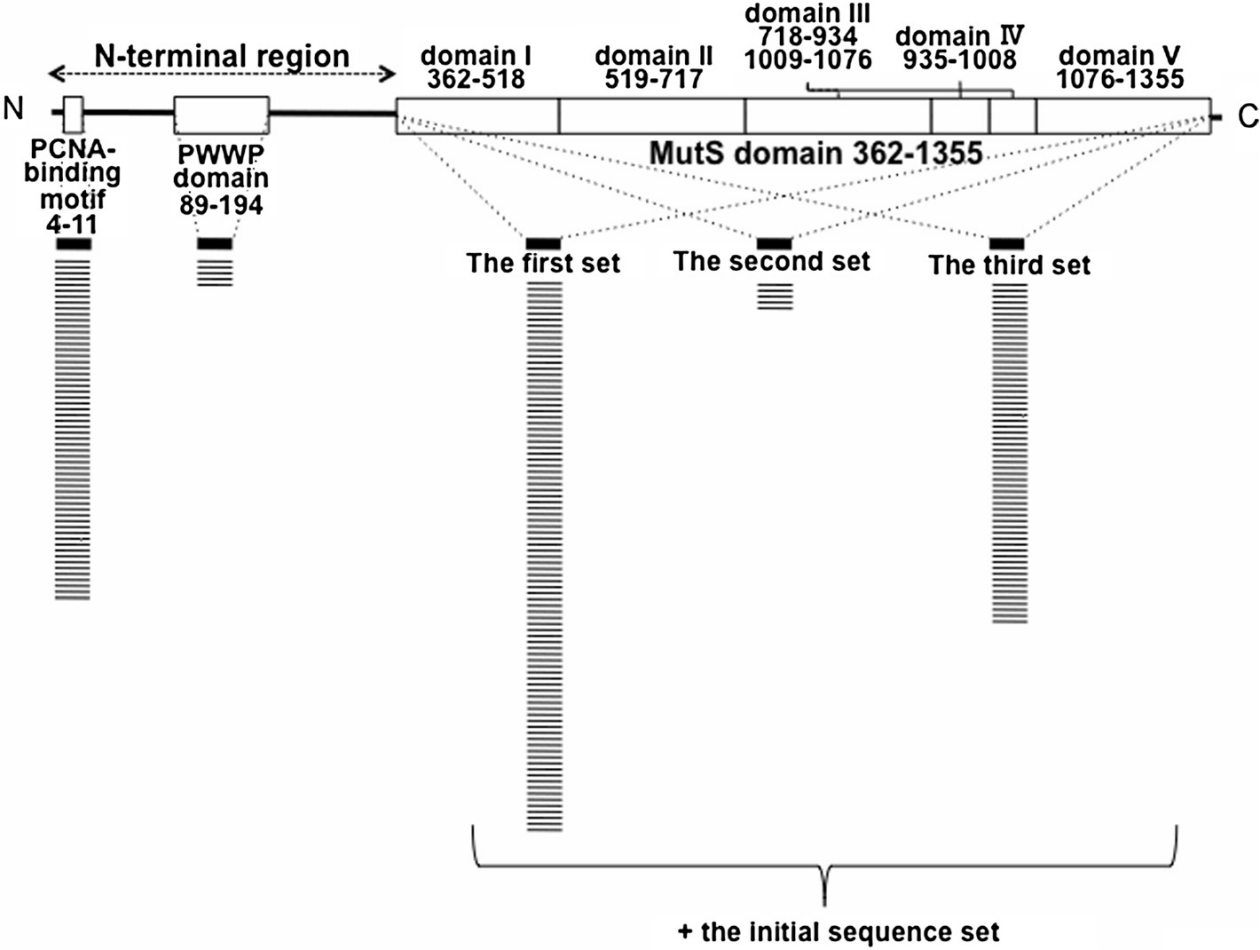
**Domain organization of human MSH6 and the additional sequence set used for optimizing MAPP parameters for MSH6.** MSH6 protein is depicted by box diagram. A box indicates a distinct domain structure and a line connecting the boxes indicates an inter-domain sequences. The range of the domain is shown above or beneath the box. “−” denotes non-vertebrate sequences in the secondary sequence set added to the initial set. For the detail, see Optimization of MAPP for MSH6 section in Results and Discussion.

We optimized MAPP [17] to predict pathogenicity of MSH6 missense variants. MAPP requires the appropriate multiple sequence alignment of MSH6 orthologues for evaluating missense variants. MSH6 amino acid sequences were collected from GenBank (http://www.ncbi.nlm.nih.gov/genbank/) using BLAST [23] by the default parameters and human MSH6 as a query sequence. The sequences were also obtained from Ensembl genome database (http://www.ensemblgenomes.org/). The inclusion of both paralogous and orthologous sequences into the multiple sequence alignment for the training of MAPP was known to worsen the performance of the prediction [14,17]. We, therefore, selected orthologues of human MSH6 sequences based on their domain organization and a phylogenetic tree. There was a wide range of variability in domain structures of the MSH6 proteins, and MSH6 sequences with the same domain organization to human MSH6 are the good candidates of orthologues. Vertebrate MSH6, the close homologues to human MSH6, generally have a PCNA-binding motif [24], a PWWP domain [25] and an MutS domain [20] (Figure 1). These vertebrate MSH6 sequences were aligned together with other MSH6 homologs by T-Coffee alignment tool [26] and a phylogenetic tree was built. This phylogenetic tree was compared with the species tree, and the proteins orthologous to human MSH6 were operationally defined by the sequences with the same domain organization that located around the human MSH6 consistently with the species tree. As a result, the vertebrate sequences were selected as an initial set and a multiple sequence alignment of them was built for MAPP prediction.

We then improved the prediction accuracy by increasing the size of the sequence set. An augmented data set was reported to improve the accuracy of the prediction [18]. The addition of amino acid sequences to the data set was limited to the domain regions, because the inter-domain sequences were too diverse to align. Sequences of non-vertebrates were added to the initial sequence set and the prediction accuracy was tested using a receiver operating characteristic (ROC) curve and the area under the curve (AUC).

### Structural properties to assess mutations in MSH6

Structural property for amino acid residue substitutions was obtained on the three-dimensional structure of MSH6-MSH2-DNA-ADP complex, registered as 2o8b [20] in Protein Data Bank [27]. The registered structure is void of residues at 551, 652, 942, and 992, and of loops at 720–728, 1099–1104, 1123–1125, 1179–1187 and 1271–1283. These missing structures were complemented using MOE (Chemical Computing Group Inc. Montreal, Canada), molecular structure building software.

Two properties we focused on were relative accessible surface area (accessibility) of each residue and the change of volumes in residues by substitution. The accessible surface area was calculated using a modified method of Shrake and Rupley [28] with water radius of 1.4 Å [29]. The threshold of 0.1 was used to separate the locations of residues into two categories; buried and surface. The relevance of accessibility to the prediction was tested based on the correlation between the accessibility and LLS/ULS. The change of volumes was quantified by the difference of the number of heavy atoms in the side chains. The relevance of this value to the prediction was also tested by the method that was the same as the one used for the accessibility test.

### Combining different properties

We used the logistic regression model to integrate the properties. The logistic regression analysis gives the probability (*q*) of a categorical variable outcome based on one or more predictor variables (*X*_*i*_). The logistic regression equation is given by: logit(*q*) = ln [*q*/(1−*q*)] = *Z* + ∑*b*_*i*_*X*_*i*_, where *Z* is the constant and *b*_*1*_*, b*_*2*_*, …, b*_*n*_ are the partial correlation coefficients for *X*_*1*_*, X*_*2*_*, …, X*_*n*_. We defined the value *q* as joint score in CoDP and this score was used for predicting the impact of UVs. The scores of MAPP for MSH6, SIFT, PolyPhen-2 and the appropriate structural properties discussed above were used as predictors *X*_*i*_. Variant sets of LLS and ULS without the biochemical functional assay were used to optimize *b*_*i*_. The applicability of the joint score for prediction was tested on the variants of LLS and ULS with the biochemical functional assay.

### Performance test

The capability of predicting the impact of UVs was tested using the variants of LLS and ULS. The prediction performance of the tools, CoDP, MAPP for MSH6, SIFT, PolyPhen-2 and PON-MMR, was compared. The comparison was carried out on prediction score distributions. The positive predictive value (PPV), the negative predictive value (NPV), sensitivity, specificity and accuracy were calculated as follows: PPV = TP / (TP + FP); NPV = TN / (FN+TN); Sensitivity = TP / (TP+FN); Specificity = TN / (FP+TN); Accuracy = (TP+TN) / (TP +TN+FP+FN), where TP is true positive, FP is false positive, TN is true negative and FN is false negative. To classify pathogenic variants, the threshold values 0.05 and 0.446 were used in SIFT [14] and PolyPhen-2 [15], respectively. The prediction performance was also compared using AUC. The box and whisker plot for each prediction was drawn to clarify the power to distinguish between LLS and ULS variants. Statistical analyses were carried out on PASW Statistics 18.0.0 software program (SPSS Inc., Chicago, IL, USA).

## Results and discussion

### The dataset of MSH6 germline missense variants

A total of 294 germline missense variants were collected from the variant databases and literature (Additional file 1: Table S1). Pathogenicity of these variants was determined based on the molecular and clinical data, and the variants were classified into three categories, namely LLS, ULS and UV (Table 1). Out of these 294 variants data, fifteen were classified as LLS (Tables 2 and 3) and nineteen as ULS (Tables 4 and 5).

**Table 2:** Variants classified as “Likely to be Lynch syndrome” (LLS) with functional assay

**No.**	**Variant**	**Definition of LLS**^***a***^	**Functional assay**					**IHC**			**MSI**	**References**
			**MMR activity**	**Interaction with MSH2**	**Locali-zation**	**ATP hydrolysis**	**Mismatch recognition**	**MLH1**	**MSH2**	**MSH6**		
1	G566R	1	Inconclusive	**Normal**	ND	**Abnormal**	ND	ND	ND	ND	H	[12,30-32]
2	R976H	1,2	ND	**Normal**	ND	ND	**Abnormal**	**Normal**	**Normal**	**Abnormal**	H	[30,33]
3	G1139S	1,2	ND	ND	ND	**Abnormal**	ND	**Normal**	Inconclusive	**Abnormal**	H	[34-36]
4	S1188N	1,2	**Abnormal**	ND	ND	ND	ND	**Normal**	**Normal**	**Abnormal**	H	[38]
5	E1193K	1,2	**Abnormal**	**Abnormal**	ND	ND	ND	**Normal**	Inconclusive	**Abnormal**	H	[31,37]

Abbreviations: *ND*, Not done, H, MSI-high.

^*a*^ See Table 1.

**Table 3:** Variants classified as LLS without functional assay

**No.**	**Variant**	**Definition of LLS**^***a***^	**IHC**			**MSI**	**Segregation study**	**FH**	**PTF**	**Healthy control =0 (N>100)**	**References**
			**MLH1**	**MSH2**	**MSH6**						
6	L449P	2,3	**Normal**	**Normal**	**Abnormal**	H	ND	**Abnormal**	**Abnormal**	ND	[39]
7	C559Y	3	ND	ND	ND	ND	**Abnormal**	**Abnormal**	**Abnormal**	ND	[44]
8	P591S	2,3	**Normal**	**Normal**	**Abnormal**	H	ND	**Abnormal**	**Abnormal**	**Abnormal**	[40]
9	P623L	3	**Normal**	**Normal**	**Abnormal**	L	ND	**Normal**	**Abnormal**	**Abnormal**	[31]
10	G670R	2	**Normal**	**Normal**	**Abnormal**	H	ND	**Normal**	**Normal**	ND	[41]
11	R772W	2	**Normal**	**Normal**	**Abnormal**	H	ND	**Normal**	**Normal**	Inconclusive (0/95)	[42]
12	Y969C	2,3	**Normal**	**Normal**	**Abnormal**	H	**Abnormal**	**Abnormal**	**Abnormal**	Inconclusive^b^	[43,44]
13	G1069E	2	**Normal**	**Normal**	**Abnormal**	H	ND	**Normal**	**Normal**	ND	[45]
14	R1076C	3	**Normal**	**Normal**	**Abnormal**	ND	ND	**Abnormal**	**Abnormal**	ND	[47,48]
15	A1236P	2,3	**Normal**	**Normal**	**Abnormal**	H	ND	**Abnormal**	NA	**Abnormal**	[46]

Abbreviations: *ND*, not done, *H*, MSI-high, *L*, MSI-low.

^*a*^ See Table 1.

^*b*^ The number of healthy population is unknown.

**Table 4:** Variants classified as “Unlikely to be Lynch syndrome” (ULS) showing normal MMR

**NO**	**Variant**	**Definition of ULS**^**a**^	**Polymorphism**	**Functional assay**					**IHC**			**MSI**	**References**
				**MMR activity**	**Interaction with MSH2**	**Localization**	**ATP hydrolysis**	**Mismatch recognition**	**MLH1**	**MSH2**	**MSH6**		
16	R128L	2	NA	**Normal**	**Normal**	ND	ND	ND	**Abnormal**	**Normal**	**Normal**	H	[31]
17	S1441	2,3	<0.01	**Normal**	**Normal**	ND	ND	ND	**Normal**	**Normal**	**Normal**	S	[30,49,50]
18	L396V	1,2	≥0.01	**Normal**	ND	ND	ND	ND	**Normal**	**Normal**	**Normal**	L/H	[32,34]
19	K728T	2,3	NA	**Normal**	**Normal**	ND	ND	ND	**Abnormal**	**Abnormal**	**Abnormal**	S	[31]

Abbreviations: *NA*, Not available, *ND*, Not done; *H*, MSI-high; *L*, MSI-low; *S*; Microsatellite stable.

^*a*^ See Table 1.

**Table 5:** Variants classified as ULS showing polymorphism or normal IHC and MSS

**No**	**Variant**	**Definition of ULS**^**a**^	**Polymorphism**	**MLH1**	**MSH2**	**MSH6**	**MSI**	**References**
**20**	**K13T**	**3**	**<0.01**	**Normal**	**Normal**	**Normal**	**S**	**[**[49]**]**
**21**	**A25V**	**1**	**≥0.01**	**ND**	**ND**	**ND**	**ND**	db S NP, 1000 Genomes
**22**	**G39E**	**1**	**≥0.01**	**ND**	**ND**	**ND**	**ND**	db S NP, 1000 Genomes
**23**	**G54A**	**3**	**NA**	**Normal**	**Normal**	**Normal**	**S**	**[**[51]**]**
**24**	**S65L**	**3**	**<0.01**	**Normal**	**Normal**	**Normal**	**S**	**[**[49]**]**
**25**	**C196F**	**1**	**≥0.01**	**ND**	**ND**	**ND**	**ND**	db S NP, 1000 Genomes
**26**	**R468H**	**3**	**<0.01**	**Normal**	**Normal**	**Normal**	**S**	**[**[49]**]**
**27**	**S503C**	**3**	**<0.01**	**Normal**	**Normal**	**Normal**	**S**	**[**[49]**]**
**28**	**R635G**	**3**	**NA**	**Normal**	**Normal**	**Normal**	**S**	**[**[52]**]**
**29**	**l886V**	**1**	**≥0.01**	**ND**	**ND**	**ND**	**ND**	1000 Genomes
**30**	**l1054F**	**3**	**NA**	**Normal**	**Normal**	**Normal**	**S**	**[**[34]**]**
**31**	**E1163V**	**1**	**≥0.01**	**ND**	**ND**	**ND**	**ND**	1000 Genomes
**32**	**E1196K**	**1**	**≥0.01**	**ND**	**ND**	**ND**	**ND**	db S NP 1000 Genomes
**33**	**E1234Q**	**1**	**≥0.01**	**ND**	**ND**	**ND**	**ND**	db S NP 1000 Genomes
**34**	**E1304K**	**1**	**≥0.01**	**ND**	**ND**	**ND**	**ND**	1000 Genomes

Abbreviations: *NA*; Not available, *ND*, Not done, S, Microsatellite stable.

^*a*^ See Table 1.

Out of fifteen LLS variants, five variants including G566R, R976H, G1139S, S1188N and E1193K showed abnormality in protein function assay (Table 2). These five variants also showed high level of MSI (MSI-H), and showed loss of MSH6 expression except for G566R variant [12,30-38]. Hence, these five variants were LLS-1 and/or LLS-2. Out of the remaining ten LLS variants (=15-5), L449P, P591S, G670G, R772W, Y969C, G1069E and A1236P variants had MSI-H and loss of MSH6 expression like the ones in Table 2, but these variants fulfilled the clinical criteria, such as family cancer history and probands’ tumor features [39-46], and hence these seven variants were LLS-2 and/or LLS-3 (Table 3). The remaining three LLS variants (=15-5-7), namely C559Y, P623L and R1076C, were LLS-3 [31,44,47,48] (Table 3).

Out of nineteen ULS variants, four variants including R128L, S144I, L396V and K728T showed normal function in protein function assay and normal staining pattern in IHC, hence fulfilled definition ULS-2 [30-32,34,49,50] (Table 4). In addition, L396V was polymorphism and also fulfilled definition ULS-1. Out of the remaining fifteen ULS variants (=19-4), K13T, G54A, S56L, R468H, S503C, R635G and I1054F variants demonstrated MSS and showed normal expression of MSH6 [34,49,51,52], hence these seven variants possessed normal MMR activity and fulfilled definition ULS-3 (Table 5). The remaining eight (=19-4-7) ULS variants, namely A25V, G39E, C196F, I886V, E1163V, E1196K, E1234Q and E1304K were polymorphism and fulfilled definition ULS-1 (Table 5).

In total, 34 variants in Tables 2, 3, 4 and 5 were available for prediction assessment, and the remaining 260 variants, which were UVs, were the targets to predict whether each of them was either LLS or ULS. In the following analyses, we used the data in Tables 3 and 5 as a parameter training data set, and the data in Tables 2 and 4 as a prediction test data set. All 34 variants data was referred to as the whole data set. And we applied the prediction to UV dataset at the end.

### Optimization of MAPP for MSH6

#### The sequence data set for the multiple alignments

From GenBank and Ensembl, 126 sequences of MSH6 orthologues were selected (Additional file 2: Table S2). Of them, 34 were derived from vertebrates. Most of the vertebrate orthologues had, from the N-terminus, a PCNA-binding motif (Qxx[LI]xx[FF], amino acid 4–11 in human MSH6) [24], a PWWP domain (amino acid 89–194) [25] and an MutS domain (amino acid 362–1355) [20] (Figure 1). These sequences were a set of initial sequences for a multiple sequence alignment.

We then added the amino acid sequences of the PCNA-binding motif and of the PWWP domain of 91 non-vertebrate MSH6 to the initial set, and found that the prediction performance was improved. The procedure of adding more amino acid sequences of MutS domain was, however, not straightforward. Three different sets of sequences were made from the non-vertebrate MutS domain. The first set contained the entire non-vertebrate MutS domain (91 sequences). The second set contained MutS domains derived from the sequences that were comprised of both the MutS and PWWP domains (5 sequences). The third set contained MutS domains derived from the sequences that were comprised of both the MutS domain and PCNA-binding motif (58 sequences). A multiple sequence alignment was built with initial sequences plus each of the described sequence sets, and the performance of prediction was tested on the whole data set using an ROC curve. The AUC of the first set was 0.767, that of the second set was 0.689 and that of the third set was 0.811. It turned out that the initial set plus the third set, namely sequences of both MutS domain and PCNA-binding motif, performed best and this set was used hereafter.

#### Normalization of the impact score

MAPP determines the pathogenicity of missense variants by an index known as impact score. The threshold of the impact score is required to determine whether the variant is pathogenic or not. The impact score basically depends on the degree of conservation of amino acid types in the alignment position [17]. Therefore, the threshold of the impact score in different domains of MSH6 likely varies. Indeed, the optimum threshold for the initial sequence set was 8.5, that for the PCNA-binding motif was 4.1, that for the PWWP domain was 5.0 and that for the MutS domain was 4.1. The different threshold values of the different domains in the same sequence could cause confusion. We, therefore, normalized the impact scores so as to make the threshold value 1.0 throughout the sequence.

#### The prediction performance of MAPP for MSH6

This type of prediction method should ideally distinguish disease-causing variants from benign variants [53]. The distributions of the score of MAPP for MSH6 between LLS and ULS variants in the whole data set were significantly different. The average for LLS and ULS was 2.673 and 0.851, respectively (Student’s *t*-test: *p* < .001) and median for LLS and ULS was 2.099 and 0.770, respectively (Mann–Whitney U test: *p* < .001). The capability of this tool is, therefore, reasonably sufficient to distinguish pathogenic variants from non-pathogenic variants.

### Development of CoDP

#### The prediction performance of SIFT and PolyPhen-2

We examined the prediction performance of both SIFT and PolyPhen-2 on the whole data set. PolyPhen-2 calculates values of both HumDiv and HumVar. HumDiv is used for diagnosis of Mendelian disease, and HumVar is used for the evaluation of rare alleles potentially involved in complex phenotypes [15]. Both SIFT and PolyPhen-2 clearly distinguished the median for LLS variants and that for ULS variants (Mann–Whitney U test: HumVar *p* < .001, HumDiv *p* < .001, SIFT *p* < .001).

#### Correlation between the structural properties of the MSH6 protein and LLS/ULS

**Figure 2:**
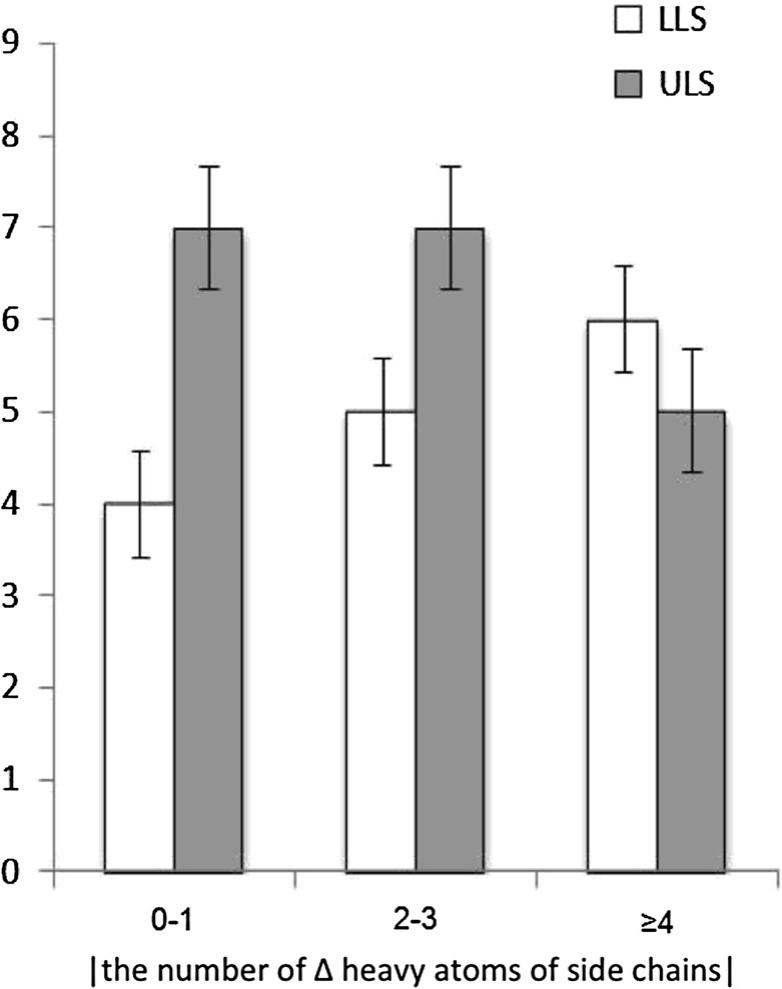
**The number of changes in heavy atoms between the original and the substituted amino acid.** For instance, in change 0–1 (no or one change in the number of heavy atoms by substitution), the cases of ULS are more frequent than those of LLS. An I-form line on each bar denotes a standard deviation obtained by the bootstrap method with 1,000 resampling. The distributions do not overlap in the number of changes 0–1 and 2–3.

The correlation between solvent accessibility of substituted amino acid and LLS/ULS was found to be statistically significant. The average of the solvent accessibility of the substituted amino acid residues in LLS and in ULS variants were 0.141 and 0.589, respectively (Student’s *t*-test: *p* < .001) and the median of the solvent accessibility of the residues in LLS and ULS variants were 0.087 and 0.583, respectively (Mann–Whitney U test: *p* < .005). The amino acid residues substituted in LLS tend to have smaller accessibility than those in ULS variants. Similarly, the correlation between the changes in the number of heavy atoms in the side chains of the substituted residues in LLS/ULS variants was also significant (Figure 2). Minor change in the number of heavy atoms in the side chains was often observed in ULS. These significant differences in the two properties evidently have a potential to be used as predictors for pathogenicity of MSH6 variants. When these two properties alone were applied to the whole data set, eleven out of 15 LLS variants and 17 out of 19 ULS variants were correctly distinguished, which is equivalent to 82.4% accuracy, using the most appropriate threshold. It is surprising to find that this simple and explicit usage of protein three-dimensional structure data had a classification power comparable to the power of SIFT and PolyPhen2.

#### Combining different properties by logistic regression model

To further improve the prediction accuracy, we combined different prediction methods above on the logistic regression equation and the weight for each method was optimized using the training data set. The logistic regression equation for joint score *q* was obtained as:

$$\begin{array}{c} \mathrm{logit}\left(q\right)=\ln\;\left[q/\left(1-q\right)\right]\\ \;=–3.7273\\ \;+0.1581\times \mathrm{the}\;\mathrm{impact}\;\mathrm{score}\;\mathrm{of}\;\mathrm{MAPP}\;\mathrm{for}\;\mathrm{MSH}6\\ \;–1.2824\times \mathrm{the}\;\mathrm{SIFT}\;\mathrm{score}\\ \;+4.6733\times \mathrm{the}\;\mathrm{PolyPhen}-2\;\left(\mathrm{HumVar}\right)\;\mathrm{score}\\ \;+1.0475\times \left|\mathrm{the}\;\mathrm{number}\;\mathrm{of}\;\Delta \;\mathrm{heavy}\;\mathrm{atoms}\;\mathrm{of}\;\mathrm{side}\;\mathrm{chains}\;\right|\\ \;–8.0548\times \mathrm{theaccessibility} \end{array}$$

The significance level is less than 1% and hence this model seems to be useful for the prediction. In the equation above, we omitted PolyPhen-2 HumDiv, because HumDiv had low accuracy, as will be explained below.

We calculated both AUC and the cut-off value of joint score *q*. AUC was 0.954 and the cut-off value was 0.56. Based on these values, we considered that the variants with the joint score *q* = 0.56 or less has minor impact on the function of the MSH6 protein, and hence the variants were likely to be non-pathogenic variants. The variants with the joint score *q* more than 0.56 were, therefore, likely to be pathogenic. More specifically, the variants with the joint score *q* more than 0.65 likely have the function impaired. And the variants with the joint score *q* between 0.56 and 0.65 likely have moderate impact on function. We applied this prediction procedure to the test data set, namely the variants with the biochemical functional assay (Tables 2 and 4), and found that the procedure predicted those variants correctly (LLS: 5/5 variants, ULS: 4/4 variants). Of the five LLS variants, four variants, namely G566R, G1139K, S1188N and E1193K, were in the category of “impaired function. ”

### Comparison of prediction performance

**Figure 3:**
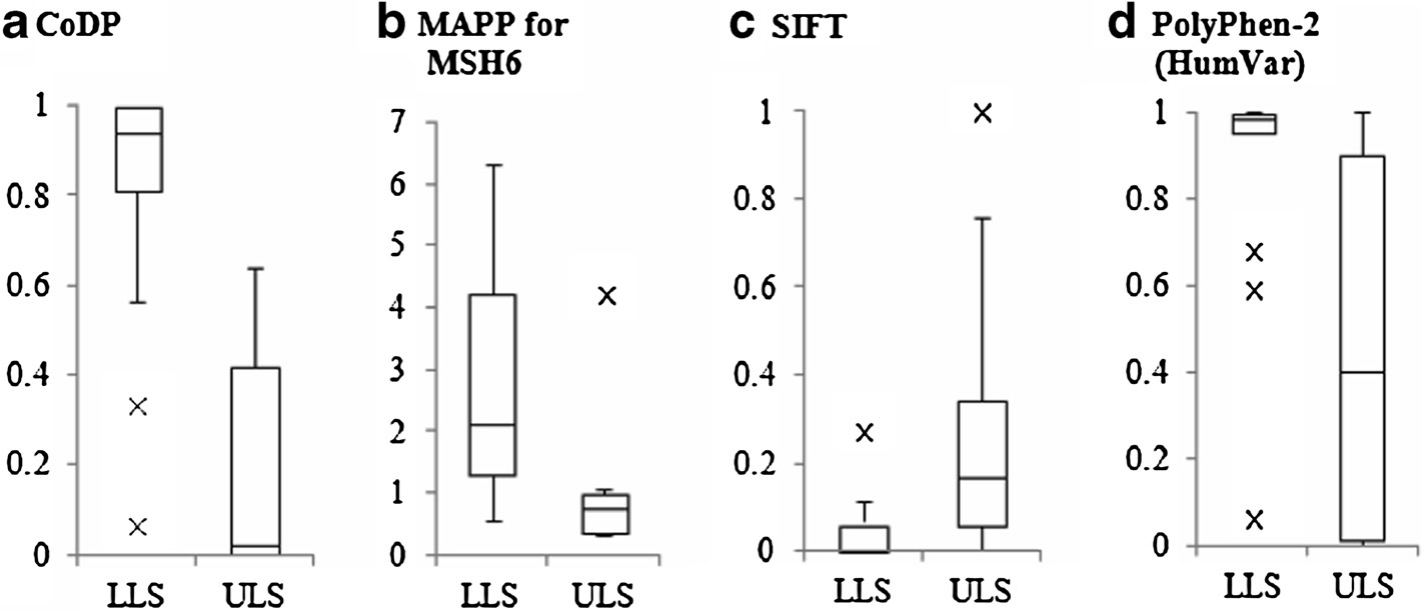
**Box and whisker plots for distributions of prediction scores of *in silico *tools in LLS and ULS variants.** The top and the bottom of the box are the 75th and 25th percentile, respectively, and the black line in the box is the median. × denotes an outlier. The distributions of LLS and ULS in CoDP (**a**) are better separated than those of MAPP for MSH6 (**b**), SIFT (**c**) and PolyPhen-2 (**d**).

The performance of CoDP was first compared with those of other conventional tools, namely MAPP, SIFT, PolyPhen-2 and PON-MMR on the whole data set. The values of PPV, NPV, sensitivity, specificity and accuracy were compared (Table 6). PPV of CoDP was 93.3% (14/15), NPV was 94.7% (18/19), sensitivity was 93.3% (14/15), specificity was 94.7% (18/19) and accuracy was 94.1% (32/34). All these scores were better than those of the conventional methods except for PON-MMR. PON-MMR predicted eleven out of 34 LLS/ULS variants as either pathogenic or non-pathogenic variants, and remaining 23 variants as UVs. The eleven variants were predicted correctly, of which three were pathogenic variants and eight were non-pathogenic variants. However, prediction by PON-MMR did not classify 23 (= 34–11) variants as pathogenic or non-pathogenic, and hence the method cannot be used for UV curation, which we aim for in our tools. Therefore, we put PON-MMR aside in this comparison. Superiority of CoDP was also clarified by AUC. AUC of CoDP was 0.954, that of MAPP for MSH6 was 0.919, of SIFT was 0.864 and of PolyPhen-2 HumVar was 0.819. The power to distinguish between LLS and ULS of these methods was visualized by the box and whisker plot (Figure 3) and further tested by Wilcoxon rank sum test. The test ended in *p* < 8.9 × 10^-6^ for CoDP, *p* < 3.3 × 10^-5^ for MAPP, *p* < 3.1 × 10^-4^ for SIFT and *p* < 1.2 × 10^-3^ for PolyPhen-2 HumVar. These tests clearly demonstrated that CoDP outperformed other conventional methods.

**Table 6:** **Prediction performance of *in silico *tools in the whole data set**

	**CoDP**	**MAPP for MSH6**	**SIFT**	**PolyPhen2 HumVar**	**PolyPhen2 HumDiv**
**TP**	**14**	**14**	**10**	**14**	**14**
**TN**	**18**	**17**	**15**	**10**	**8**
**FP**	**1**	**1**	**4**	**9**	**11**
**FN**	**1**	**2**	**5**	**1**	**1**
**PP0V**	**0.933 (14/15)**	**0.875 (14/16)**	**0.714 (10/14)**	**0.609 (14/23)**	**0.560 (14/25)**
**NPV**	**0.947 (18/19)**	**0.944 (17/18)**	**0.750 (15/20)**	**0.909 (10/11)**	**0.889 (8/9)**
**Sencitivity**	**0.933 (14/15)**	**0.875 (14/15)**	**0.667 (10/15)**	**0.933 (14/15)**	**0.933 (14/15)**
**Specificity**	**0.941 (32/34)**	**0.912 (31/34)**	**0.735 (25/34)**	**0.706 (24/34)**	**0.647 (22/34)**

When the performances of the tools were compared on the test data set alone, only CoDP predicted all test variants correctly. The values of PPV, NPV, sensitivity, specificity and accuracy of the tools in the test data set were shown in Table 7 (MAPP LLS: 4/5 variants, ULS: 4/4 variants; SIFT LLS: 4/5 variants, ULS: 4/4 variants; PolyPhen-2 HumVar LLS: 5/5 variants, ULS: 2/4 variants). AUC of CoDP was 1.000, that of MAPP for MSH6 was 0.800, of SIFT was 0.950 and of PolyPhen-2 HumVar was 0.900. The power to distinguish between LLS and ULS of these methods on the test data set was *p* < 1.5 × 10^-2^ for CoDP, *p* < 1.9 × 10^-1^ for MAPP, *p* < 6.5 × 10^-2^ for SIFT and *p* < 1.5 × 10^-2^ for PolyPhen-2 HumVar. The box and whisker plot that visualized the distribution of the scores were shown in Additional file 3: Figure S1.

**Table 7:** **Prediction performance of *in silico *tools in the test set**

	**CoDP**	**MAPP for MSH6**	**SIFT**	**PolyPhen2 HumVar**	**PolyPhen2 HumDiv**
**TP**	**5**	**4**	**4**	**5**	**5**
**TN**	**4**	**4**	**4**	**2**	**1**
**FP**	**0**	**0**	**0**	**2**	**3**
**FN**	**0**	**1**	**1**	**0**	**0**
**PPV**	**5/5**	**4/4**	**4/4**	**5/7**	**5/8**
**NPV**	**4/4**	**4/5**	**4/5**	**2/2**	**1/0**
**Sencitivity**	**5/5**	**4/5**	**4/5**	**5/5**	**5/5**
**Specificity**	**4/4**	**4/4**	**4/4**	**2/4**	**1/4**
**Accuracy**	**9/9**	**8/9**	**8/9**	**7/9**	**6/9**

The small size of the test data set may raise doubts on the superiority of CoDP. To overcome the paucity of the test sample, we also employed a leave-one-out jackknife method and evaluated the performance of the tools. CoDP predicted 85.3% (29/34, LLS 93.3%, 14/15, ULS 78.9%, 15/19) of the variants correctly and the performance was still better than SIFT and PolyPhen-2 HumVar (Table 6). Here, we did not compared the performance of CoDP and MAPP for MSH6, because of the fact that MAPP is based on the information retrieved from the homologous sequences and hence it was difficult to leave the information of the target sequence out of the training set.

### Predicting UVs by CoDP

We now used CoDP to interpret 260 germline missense variants, which were classified as UVs. Of 260 UVs, 84 variants (32.3%) were predicted as pathogenic variants, and 176 variants (67.7%) as non-pathogenic variants, hence about one third of the UVs detected in MSH6 were predicted as pathogenic variants. Of these putative 84 pathogenic variants, three variants were predicted to have the moderate impact on the protein (0.56 < joint score *q* ≤ 0.65), and the 81 variants were predicted to have impaired function (joint score *q* > 0.65) (Table 8).

**Table 8:** Classification results of UVs in MSH6 by CoDP

**The variants with no impact on MSH6**						**The variants with moderate impact on MSH6**		**The variants with impact on MSH6**			
**Variants**	**Score**	**Variants**	**Score**	**Variants**	**Score**	**Variants**	**Score**	**Variants**	**Score**	**Variants**	**Score**
S9G	0.000	S360I	0.000	L815I	0.180	G670V	0.595	L370S	0.832	A1021D	0.988
A20V	0.000	R361H	0.000	P831A	0.060	S1049F	0.572	Y397C	0.976	R1024W	0.938
A20D	0.000	T369I	0.009	D857N	0.426	I1227L	0.619	L435P	0.942	D1026Y	0.995
**N21S**	**0.000**	**E381K**	**0.001**	**V867G**	**0.189**			**A457P**	**0.951**	**D1031V**	**0.722**
**A25S**	**0.000**	**D390N**	**0.003**	**V878A**	**0.009**			**R468C**	**0.992**	**R1034Q**	**0.724**
**A36V**	**0.000**	**Y397F**	**0.003**	**D880E**	**0.000**			**V474A**	**0.930**	**A1055T**	**0.935**
**P42S**	**0.000**	**I425V**	**0.115**	**Q889H**	**0.022**			**V480L**	**0.853**	**D1058S**	**0.975**
**W50R**	**0.000**	**I442T**	**0.017**	**I891M**	**0.031**			**E484K**	**0.826**	**V1059A**	**0.716**
**A81T**	**0.000**	**E446N**	**0.027**	**L893V**	**0.016**			**V509A**	**0.969**	**A1064V**	**0.846**
**A81V**	**0.000**	**N455T**	**0.000**	**R901H**	**0.035**			**I516N**	**0.740**	**Y1066C**	**0.999**
**K99N**	**0.003**	**Q475H**	**0.261**	**D904E**	**0.006**			**T521I**	**0.911**	**P1087H**	**0.978**
**I120V**	**0.000**	**K476E**	**0.145**	**V907A**	**0.001**			**Y535C**	**0.894**	**P1087R**	**0.995**
**E122K**	**0.000**	**M492V**	**0.530**	**E983Q**	**0.074**			**Y538S**	**0.998**	**R1095H**	**0.692**
**K125E**	**0.000**	**R497T**	**0.028**	**N984H**	**0.006**			**D575Y**	**0.997**	**R1095C**	**0.996**
**L147H**	**0.000**	**K498R**	**0.000**	**F985L**	**0.016**			**S580L**	**0.997**	**T1100R**	**0.860**
**A159V**	**0.000**	**Q522R**	**0.097**	**R988L**	**0.017**			**P656L**	**0.943**	**I1115T**	**0.802**
**H164P**	**0.000**	**P531T**	**0.003**	**P991L**	**0.065**			**S682C**	**0.653**	**T1142M**	**0.864**
**K185E**	**0.000**	**E533D**	**0.006**	**T1008I**	**0.302**			**S682F**	**0.998**	**G1148R**	**1.000**
**K187T**	**0.000**	**E546G**	**0.031**	**R1024Q**	**0.053**			**G685A**	**0.939**	**G1157S**	**0.964**
**E192V**	**0.000**	**E546Q**	**0.003**	**Q1048E**	**0.002**			**L700F**	**0.985**	**A1162P**	**0.970**
**V195F**	**0.015**	**S549F**	**0.468**	**V1056M**	**0.360**			**S702G**	**0.951**	**T1175S**	**0.822**
**D197H**	**0.001**	**Y556F**	**0.162**	**R1068G**	**0.312**			**F706S**	**0.996**	**E1187G**	**0.998**
**E198A**	**0.000**	**I570V**	**0.054**	**P1073S**	**0.001**			**R761G**	**0.922**	**L1201F**	**0.984**
**P202A**	**0.000**	**R577H**	**0.522**	**P1073R**	**0.042**			**C765W**	**1.000**	**D1213V**	**0.932**
**M208V**	**0.000**	**F582L**	**0.146**	**V1078A**	**0.004**			**G770V**	**0.994**	**E1214A**	**0.992**
**V210A**	**0.000**	**I608V**	**0.033**	**P1082S**	**0.018**			**R772Q**	**0.954**	**R1217K**	**0.880**
**V215I**	**0.000**	**K610N**	**0.009**	**P1082L**	**0.012**			**W777R**	**0.994**	**T1219I**	**0.944**
**D217Y**	**0.001**	**E619D**	**0.291**	**P1087T**	**0.056**			**A780G**	**0.713**	**T1225M**	**0.888**
**E220D**	**0.000**	**P623A**	**0.010**	**P1087S**	**0.201**			**I795T**	**0.707**	**R1242L**	**0.966**
**E221D**	**0.000**	**G624S**	**0.072**	**E1090K**	**0.007**			**L798V**	**0.919**	**T1243S**	**0.650**
**N223D**	**0.000**	**E639K**	**0.005**	**T1100M**	**0.025**			**Y850C**	**1.000**	**V1253E**	**0.856**
**N223S**	**0.000**	**R644S**	**0.057**	**K1101N**	**0.002**			**K854M**	**0.826**	**R1263C**	**0.767**
**S227I**	**0.000**	**K646R**	**0.223**	**P1110S**	**0.376**			**S860F**	**0.982**	**R1263H**	**0.669**
**E229G**	**0.008**	**I651T**	**0.000**	**I1113T**	**0.045**			**K866T**	**0.685**	**M1267T**	**0.946**
**P233R**	**0.000**	**M654I**	**0.001**	**E1121D**	**0.000**			**Q889P**	**0.682**	**C1275Y**	**0.992**
**R243C**	**0.005**	**S666P**	**0.008**	**A1151V**	**0.055**			**L909S**	**0.967**	**T1284M**	**0.913**
**R243H**	**0.000**	**D667H**	**0.453**	**V1160I**	**0.117**			**D943Y**	**0.900**	**A1303T**	**0.981**
**I245L**	**0.000**	**I669T**	**0.000**	**D1181E**	**0.540**			**Y977H**	**0.945**	**A1303G**	**0.916**
**I251V**	**0.000**	**P673A**	**0.405**	**M1202V**	**0.009**			**R988C**	**0.716**	**R1321G**	**0.825**
**I258T**	**0.000**	**E675D**	**0.000**	**V1232L**	**0.318**			**Y994H**	**0.895**	**L1353W**	**0.989**
**F265C**	**0.119**	**K676R**	**0.006**	**H1248D**	**0.022**			**S998T**	**0.853**		
**T269S**	**0.000**	**Q698K**	**0.005**	**V1253L**	**0.068**						
**K270M**	**0.001**	**Q698E**	**0.006**	**V1260I**	**0.001**						
**E277D**	**0.000**	**A704G**	**0.008**	**N1273S**	**0.008**						
**S285I**	**0.000**	**T719I**	**0.006**	**E1274K**	**0.006**						
**G289D**	**0.000**	**T720A**	**0.033**	**S1279P**	**0.014**						
**G289E**	**0.000**	**T720I**	**0.024**	**I1283V**	**0.001**						
**K295E**	**0.000**	**I725M**	**0.000**	**E1310D**	**0.001**						
**K295R**	**0.001**	**I725V**	**0.000**	**E1311D**	**0.004**						
**R300P**	**0.001**	**F726S**	**0.208**	**R1321S**	**0.128**						
**S314I**	**0.000**	**R761K**	**0.015**	**M1326I**	**0.001**						
**S314R**	**0.001**	**T764N**	**0.005**	**M1326T**	**0.002**						
**S315F**	**0.003**	**P768A**	**0.201**	**S1329L**	**0.014**						
**T319M**	**0.000**	**C783S**	**0.409**	**R1331L**	**0.011**						
**P320T**	**0.000**	**A787V**	**0.063**	**R1334Q**	**0.000**						
**A326V**	**0.000**	**V800L**	**0.000**	**D1346N**	**0.001**						
**T327S**	**0.000**	**V800A**	**0.000**	**L1354Q**	**0.018**						
**F340S**	**0.001**	**D803G**	**0.003**	**K1358E**	**0.001**						
**S360G**	**0.000**	**S806F**	**0.450**								

The higher joint scores of CoDP tend to derive from the mutations in the conserved domain, namely in the MutS domain. This tendency suggests that missense mutations in the domain should have considerable influence on protein function. The MutS domain in MSH6 forms a heterodimer with MSH2 and participates in the early recognition of mismatches and small insertion/deletion loops of DNA [54,55]. For instance, the E1193K variant, classified as LLS, is located in the MutS domain V region (Figure 1). The MutS domain V region is the highly conserved region in MutS homologues [20]. This variant showed remarkable impairment of function, such as the loss of heterodimerization with MSH2 and MMR activity [31]. CoDP gave the joint score *q* = 0.813 to E1193K variant, indicating that the variant likely has significant damage to the structure of MSH6, which may impair the function of the protein.

## Conclusion

In this study, we built CoDP, the new prediction tool to assess the MSH6 missense variants. The novelty of CoDP lies in the direct incorporation of protein three-dimensional structure information and the introduction of the logistic regression model for combining the different prediction methods. The former feature was found to have unexpectedly high performance in LLS/ULS classification, and the latter procedure can be interpreted as an introduction of a simple neural network model for combining outputs from different prediction schemes. These new features enabled CoDP to achieve better performance for the classification of the MSH6 variants. The better performance was also sustained by the manually curated dataset of MSH6 variants presented in Tables 2, 3, 4, 5, and 6.

For adjusting the parameters, we carefully categorized MSH6 germline missense variants into LLS and ULS. In the current dataset, only 34 out of 294 variants could be categorized into LLS and ULS. This was due to the paucity of both biochemical functional assay data and clinical and molecular data that are linked to the variants of MSH6 on the databases. This data paucity makes the present CoDP not be clinically applicable. However, current form of CoDP has better utility for supporting a risk estimation of UVs in MSH6, as SIFT or PolyPhen-2 does to other proteins. In the future when more associated data would be obtained, the appropriate parameters would be set, and the accuracy of CoDP would be further improved.

## Abbreviations

AUC: The area under the curve; CRC: Colorectal cancer; HNPCC: Hereditary Non-Polyposis Colorectal Cancer; IHC: Immunohistochemistry; LLS: Likely to be Lynch syndrome; MAPP: Multivariate analysis of protein polymorphisms; MMR: Mismatch repair; MSI: Microsatellite instability; MSI-H: High level of microsatellite instability; MSI-L: Microsatellite instability low; MSS: Microsatellite stable; NPV: The negative predictive value; PPV: The positive predictive value; ROC: A receiver-operating characteristic; ULS: Unlikely to be Lynch syndrome; UVs: Unclassified variants

## Competing interests

The authors declare that they have no competing interests.

## Authors’ contribution

HT performed the majority of the work presented in this manuscript and drafted the manuscript. HT, KA and KY participated in this research. HK assisted in research carried out. All authors read and approved the final manuscript.

## Supplementary Material

Additional file 1: Table S1 MSH6 missense variants data used for parameter fitting. The file can be read by standard TIF viewer, such as Preview on Mac OS X. Click here for file

Additional file 2: Table S2 A list of amino acid sequences used for the multiple sequence alignment of MSH6. The file can be read by standard TIF viewer, such as Preview on Mac OS X. Click here for file

Additional file 3: Figure S1 Box and whisker plots for the score distribution of *in silico* tools evaluated on the test set. The top and the bottom of the box are the 75th and 25th percentile, respectively, and the white line in the box is the median. The distributions of LLS and ULS are divided clearly. The file can be read by standard TIF viewer, such as Preview on Mac OS X. Click here for file
