# Supplementary figures and images for: CoDP: predicting the impact of unclassified genetic variants in MSH6 by the combination of different properties of the protein
Source: J Biomed Sci. 2013 Apr 28;20(1):25. doi: 10.1186/1423-0127-20-25 (PMC3651391; doi:10.1186/1423-0127-20-25)

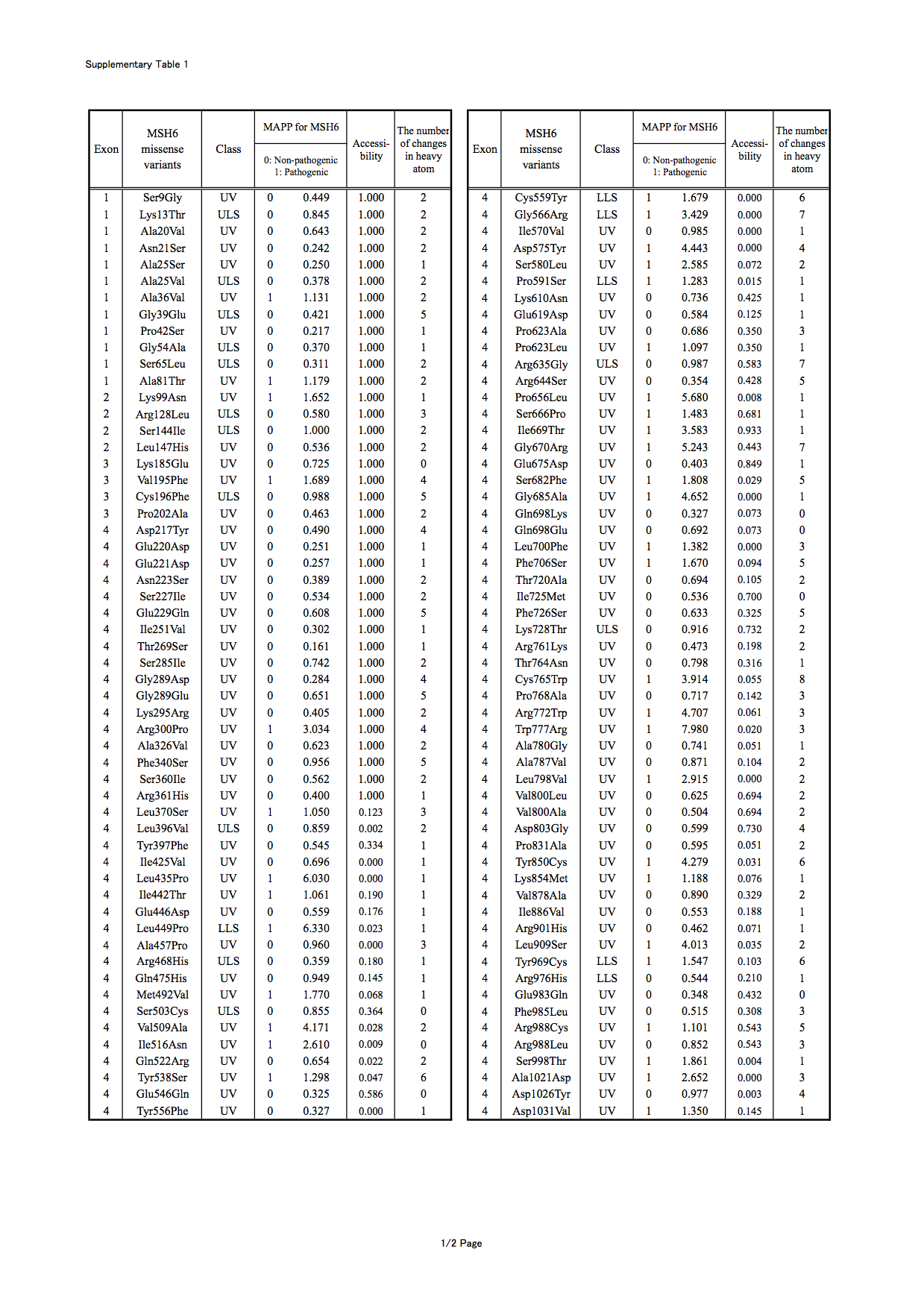

Supplement: Additional file 1: Table S1 — MSH6 missense variants data used for parameter fitting. The file can be read by standard TIF viewer, such as Preview on Mac OS X. [file 1423-0127-20-25-S1.tiff]

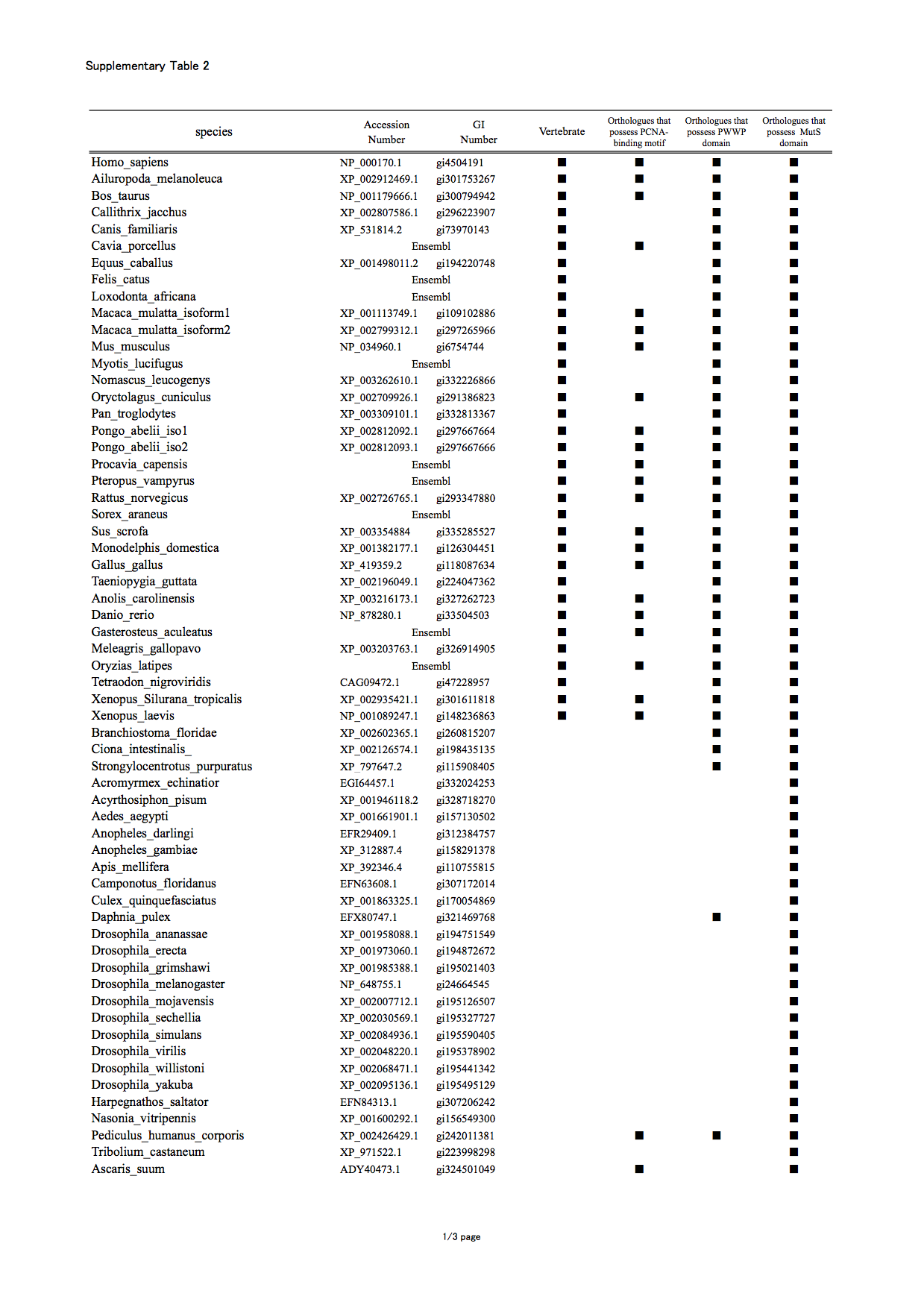

Supplement: Additional file 2: Table S2 — A list of amino acid sequences used for the multiple sequence alignment of MSH6. The file can be read by standard TIF viewer, such as Preview on Mac OS X. [file 1423-0127-20-25-S2.tiff]

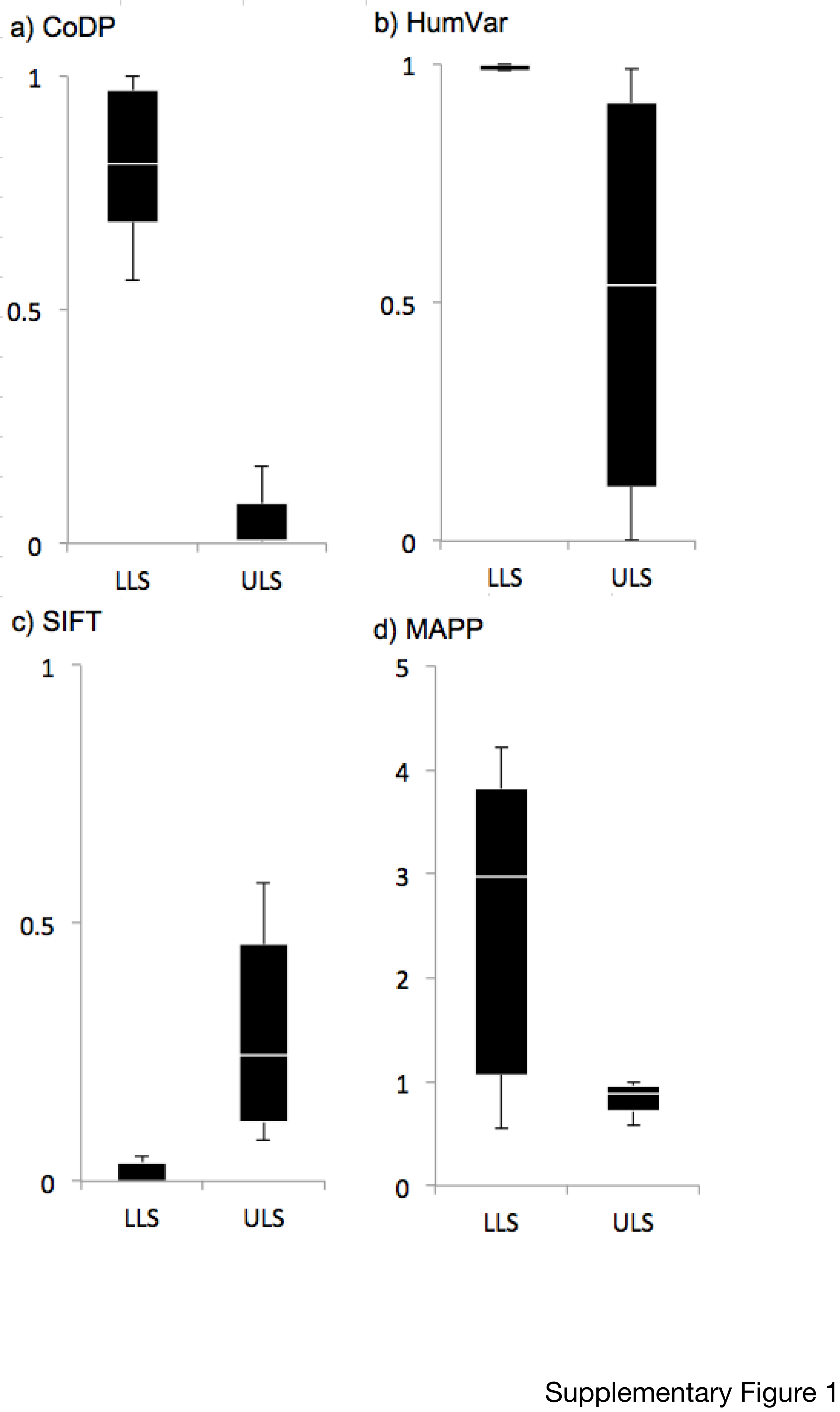

Supplement: Additional file 3: Figure S1 — Box and whisker plots for the score distribution of in silico tools evaluated on the test set. The top and the bottom of the box are the 75th and 25th percentile, respectively, and the white line in the box is the median. The distributions of LLS and ULS are divided clearly. The file can be read by standard TIF viewer, such as Preview on Mac OS X. [file 1423-0127-20-25-S3.tiff]
